# The Relationship between Theory of Mind and Intelligence: A Formative *g* Approach

**DOI:** 10.3390/jintelligence9010011

**Published:** 2021-02-19

**Authors:** Ester Navarro, Sara Anne Goring, Andrew R. A. Conway

**Affiliations:** Division of Behavioral and Organizational Sciences, School of Social Science, Policy and Evaluation, Claremont Graduate University, Claremont, CA 91711, USA; sara.goring@cgu.edu (S.A.G.); andrew.conway@cgu.edu (A.R.A.C.)

**Keywords:** general intelligence, theory of mind, factor analysis, structural equation modeling

## Abstract

Theory of Mind (ToM) is the ability understand that other people’s mental states may be different from one’s own. Psychometric models have shown that individual differences in ToM can largely be attributed to general intelligence (*g*) (Coyle et al. 2018). Most psychometric models specify *g* as a reflective latent variable, which is interpreted as a general ability that plays a causal role in a broad range of cognitive tasks, including ToM tasks. However, an alternative approach is to specify *g* as a formative latent variable, that is, an overall index of cognitive ability that does not represent a psychological attribute (Kovacs and Conway 2016). Here we consider a formative *g* approach to the relationship between ToM and intelligence. First, we conducted an SEM with reflective *g* to test the hypothesis that ToM is largely accounted for by a general ability. Next, we conducted a model with formative *g* to determine whether the relationship between ToM and intelligence is influenced by domain-specific tasks. Finally, we conducted a redundancy analysis to examine the contribution of each *g* variable. Results suggest that the relationship between ToM and intelligence in this study was influenced by language-based tasks, rather than solely a general ability.

## 1. Introduction

Theory of mind (ToM) is a psychological construct that reflects the ability to attribute thoughts, feelings, and beliefs to others (Premack and Woodruff 1978; Wimmer and Perner 1983). ToM is necessary to navigate complex social interactions and is considered a higher-order ability that requires a number of underlying processes, some domain-general and some domain-specific (a higher-order cognitive ability is one that relies on multiple lower-order processes for functioning (e.g., WMC is a lower-order process that combined with other processes produces the higher-order ability “reading comprehension”)) (Quesque and Rossetti 2020). For example, ToM requires working memory and cognitive control (domain-general) as well as linguistic reasoning (domain-specific). Prior research indicates that ToM task performance is related to a number of cognitive abilities, such as executive function (Carlson and Moses 2001), language ability (Milligan et al. 2007), and IQ (e.g., Baker et al. 2014; Dodell-Feder et al. 2013). Although research has established that ToM is related to multiple cognitive abilities, the relationship between ToM and intelligence remains unclear.

In psychometrics, many researchers interpret the general factor (*g*) derived from shared variance among cognitive tests as a general intelligence or general ability, that is, a cognitive ability that plays a causal role in performance of mental tasks. This view was developed as an explanation for the *positive manifold* (Carroll 1993; Spearman 1904), that is, the finding that all tests of cognitive ability are positively correlated. *g* is thought to be responsible for the positive manifold because it represents the shared variance among a number of cognitive tests (Conway and Kovacs 2013, 2018). In other words, this approach characterizes *g* as a psychological attribute with a reflective property (i.e., an underlying causal influence on all fundamental cognitive processes). Since all tests of cognitive ability are positively correlated and ToM is positively correlated with multiple cognitive abilities, some researchers have proposed that individual differences in ToM can be largely explained by *g* and are, therefore, caused by a general cognitive ability (Coyle et al. 2018).

However, the claim that ToM can be accounted for by a general cognitive ability is inconsistent with the ToM literature and most theoretical frameworks of ToM. For example, research has shown that different underlying higher-order abilities (e.g., causal inference) and lower-order processes (e.g., gaze tracking) are needed for different ToM tasks (Schaafsma et al. 2015) and different brain areas are involved in responses to ToM tasks. If successful ToM requires the use of several different processes, some domain-general and some domain-specific, then it is unlikely that general ability alone can explain all, or even most of, the variation in ToM task performance.

While reflective models consider *g* to represent general ability and the underlying cause of all other abilities, formative theories of *g* might provide a more accurate account of the relationship between ToM and intelligence. Formative models propose that *g* is an emergent property and the result of multiple cognitive processes that are sampled in an overlapping manner across a battery of cognitive tasks (Kovacs and Conway 2016). The relationship between ToM and intelligence has been studied from the perspective of reflective *g* (Coyle et al. 2018), but it has not been examined from the perspective of formative *g*.

The purpose of the current study is to offer an alternative perspective on the relationship between ToM and intelligence. We will contrast reflective *g* and formative *g* models to determine whether they provide similar or different accounts of the relationship between ToM and intelligence.

### 1.1. The Cognitive and Neural Underpinnings of Theory of Mind

Theory of mind (ToM) is the ability to understand the beliefs, knowledge, and intentions of others based on their behavior. The term was first coined by Premack and Woodruff to refer to chimpanzees’ ability to infer human goals, and it was quickly adopted by psychologists to study humans’ ability to infer and predict the behavior of others. This was followed by a vast number of studies on the topic. A simple search of the term “theory of mind” on PsycInfo reveals over 7000 articles and 1000 books on Theory of Mind. This is not surprising given that ToM is necessary for numerous complex cognitive tasks, including communication (e.g., Grice 1989; Sperber and Wilson 1995), criticism (Cutting and Dunn 2002), deception (Sodian 1991), irony (Happé 1994), pragmatic language competence (Eisenmajer and Prior 1991), aggressive behavior (Happé and Frith 1996), and problem solving (Greenberg et al. 2013).

A large amount of research originally focused on studying children’s development of ToM, specifically related to the age at which children developed this skill (see Wellman et al. 2001 for a meta-analysis), and presumed that adults’ ToM was largely a fully-fledged skill (Keysar et al. 2003). However, research soon showed that adults also fail to use their ToM in some circumstances, such as when their perspectives differ from the other person’s perspective (e.g., Apperly et al. 2008; Dumontheil et al. 2010; Keysar et al. 2003; Rubio-Fernández and Glucksberg 2012) or when a person has privileged information (e.g., Epley et al. 2004; Mitchell et al. 1996; Navarro et al. 2020), suggesting that even if adults’ ToM is more advanced than children’s ToM, there are still individual differences in the extent to which adults can use ToM effectively. In addition, research has shown that different specific regions within the so-called ToM network (including the medial prefrontal cortex, and the left and right temporoparietal junction; Gallagher and Frith 2003; Saxe et al. 2004) are utilized at different developmental stages, reflecting changes in the way ToM is used across the lifespan (Bowman and Wellman 2014). For example, in infancy, regions engaged in ToM tend to be more diffuse (i.e., more areas are activated); however, there is a gradual incorporation of regions in the ToM network and a shift in the type of functions used as development proceeds (Bowman and Wellman 2014). This suggests that changes that occur in infancy could influence later development, and therefore ToM development does not necessarily end in early childhood.

Given the differences between children and adults’ ToM, to effectively measure ToM in adults, a large number of tasks have been developed over the years (for a review, see Quesque and Rossetti 2020) that assess individuals’ ability to interpret other people’s intentions, perspectives, and emotions. For this reason, it is advisable to use more than one task to better tap into a general ToM construct (Baker et al. 2014; Rice-Warnell and Redcay 2019).

Despite the general consensus that adult ToM is an area worth examining, there is still ample debate on the mechanisms responsible for ToM. Several researchers have proposed that ToM might depend on different modules or processes (Leslie 1994; Leslie and Polizzi 1998; Apperly and Butterfill 2009). For example, Leslie proposed that a specific ToM mechanism (ToMM) is responsible for responding to domain-specific aspects of a task, while a general selection processor is needed to overcome effortful aspects of ToM, such as inhibiting one’s own perspective. Similarly, Gopnik et al. (1997) proposed that ToM has a strong experience-dependent component, needed to achieve appropriate comprehension of the mental states of others. These relevant theories have in common the fact that they propose a combination of domain-general and domain-specific processes needed to engage ToM. Neuroimaging and psychometric research further support the idea that ToM requires both context-specific information and domain-general cognitive processes (Wellman 2018). Specifically, the ToM network comprises a number of neural regions that are activated during tasks that require ToM, such as perspective taking (Frith and Frith 2003; Schurz et al. 2014), but not during control tasks. Further, different areas of the ToM network are especially engaged on some types of ToM tasks, but not others (see Schaafsma et al. 2015). This indicates that ToM is a flexible construct that depends on a number of brain areas. For this reason, most ToM accounts suggest that ToM involves a multicomponent system formed by a number of interdependent domain-general processes, such as general perspective-taking and inhibition, as well as domain-specific processes, such as language, emotional processing (Oakley et al. 2016), and automatic eye movements (Obhi 2012). Thus, this research suggests that it is not likely that ToM is solely dependent on a general cognitive ability.

### 1.2. The Study of Intelligence and g

There is a long tradition of submitting cognitive test scores to factor analysis in order to extract a single common factor, *g*, representing general intelligence (Spearman 1904, 1927). With the revelation that cognitive abilities are consistently positively correlated (i.e., the positive manifold), many researchers interpreted *g* as the common cause underlying individual differences in task performance and the covariance among different measures (Gottfredson 1997). Although this viewpoint has dominated intelligence research for over a century, theoretical accounts of intelligence based on reflective *g* struggle to integrate evidence from psychometrics, cognitive psychology, and neuroscience, despite many years of research attempting to test various theories using traditional factor analysis techniques (see Kovacs and Conway 2019).

Spearman first proposed the reflective view that *g* is an underlying latent variable that causes the correlations observed among cognitive tasks, regardless of the domain-specific subject matter assessed in the task (Jensen 1998). However, alternative explanations for the positive associations amongst cognitive tasks have begun to emerge as researchers reassess the validity of general ability (*g*) being the cause of the positive manifold. Specifically, formative models posit that there is not a unitary cause underlying the positive manifold, rather the finding of a general factor is merely a mathematical consequence of multiple cognitive processes sampled across a battery of tasks in an overlapping fashion (Kovacs and Conway 2016). Thus, the correlation between any two tasks reflects the shared processes sampled by those tasks. This is what Process Overlap Theory proposes (POT; Kovacs and Conway 2016). POT provides an account of the hierarchical structure of intelligence and the relationship between *g* and other broad cognitive abilities (e.g., verbal ability, spatial ability) without positing a causal general ability (Conway et al. 2020). According to POT, *g* results from the overlapping domain-general executive attention processes and domain-specific processes sampled across a battery of tasks, with domain-general processes being sampled across a broader range of tasks and with higher probability than domain-specific processes. Importantly, this implies that cognitive tasks are never process-pure. Instead, multiple domain-general and multiple domain-specific processes are necessary for cognitive functioning. This is important because reflective *g* capitalizes on shared variance across tasks, leading to approaches that partition domain-general from domain-specific variance to determine the source of covariance in the data (Coyle et al. 2018). However, the formative *g* view is derived from aggregating the shared variance across tasks, and therefore, the different sources of variance cannot and should not be partitioned from one another. As such, formative approaches to *g,* such as POT, attempt to explain intelligence as more complex than solely the result of a general ability that causes variance in performance on all tests.

This is especially relevant when trying to explain the relationship between intelligence and ToM. For example, taking a reflective *g* perspective, research has reported that the relationship between ToM and intelligence can be explained almost entirely by general ability. Specifically, Coyle et al. (2018) examined the relationship between ToM and intelligence using structural equation modeling (SEM). Coyle et al. (2018) hypothesized that the relationship between ToM and executive function likely indicates that ToM is also largely dependent on and could be almost entirely explained by general ability. To this end, Coyle et al. (2018) examined the causal relationship between scores from three subtests of the ACT (English, Math, and Reading) and two ToM tasks. In addition, the residuals of the ACT and ToM tests were used as indicators of domain-specific variability (i.e., non-*g* residuals). According to Coyle et al. (2018), if ToM reflects an ability separate from general intelligence, then ToM should be partially predicted by the non-*g* residuals. In turn, if ToM is largely dependent on general ability, then ToM should be fully predicted by *g*. According to Coyle et al. (2018), previous research indicates that non-*g* residuals are predictive of domain-specific cognitive outcomes (e.g., math test; Coyle et al. 2013) and therefore could be used to predict non-*g*-specific variance in ToM. The model proposed by Coyle et al. (2018) found a strong predictive relationship between *g* and ToM, whereas the non-*g* residuals were not related to ToM. This led Coyle et al. (2018) to conclude that individual differences in ToM are due to “not much more than *g*” (Coyle et al. 2018, p. 90).

However, Coyle et al. (2018) based their hypothesis on the reflective view of *g*. Based on research suggesting that ToM is influenced by both domain-general and domain-specific processes (see Wellman 2018), a formative *g* model might provide a more accurate account of the relationship between ToM and intelligence.

### 1.3. The Current Study

The current study examined the relationship between ToM and intelligence by taking a formative *g* perspective. Coyle et al. (2018) proposed that general ability is the cause of ToM based on the lack of correlation between ToM and non-*g* residuals. However, this is based on the assumption that non-*g* residuals reflect a domain-specific ability (Coyle 2014). Formative *g* models may better explain the relationship between ToM and intelligence because this view does not assume that domain-general and domain-specific processes can be partitioned, and instead facilitates the exploration of the individual contribution that each variable makes to the latent factor.

For this purpose, we examined a large dataset (Freeman et al. 2019), that contained the original data used by Coyle et al. (2018), as well as new data, to examine the relationship between ToM and intelligence from a formative *g* perspective. Specifically, in this study, we first conducted a confirmatory factor analysis (CFA) and structural equation model (SEM) with reflective *g* to replicate Coyle et al.’s original models. Next, we conducted an SEM, based on a formative interpretation of *g*, to examine the extent to which the relationship between ToM and intelligence is dependent on language ability. Finally, we conducted a redundancy analysis to examine the contribution of each of the *g* variables to ToM without the need of an overarching construct.

## 2. Assessing the Relationship between *g* and ToM

The goal of this study was to assess the hypothesis that ToM is largely attributed to a general ability, *g* (Coyle et al. 2018). For this purpose, we first conducted a confirmatory factor analysis (CFA) with reflective *g* to confirm the fit of the measurement model, followed by an SEM with a predictive path between reflective *g* and ToM. Next, we conducted structural models, using a formative *g* in the base measurement model, to assess the relationship between ToM and intelligence from a formative *g* perspective. Specifically, we conducted a formative-*g* SEM, with all ACT measures contributing to the latent variable. We also conducted additional formative-*g* models: in each of which, the factor loadings of one of the ACT subtests was constrained to zero to assess the necessity and contribution of the individual measures. This approach allowed us to determine whether the relationship between *g* and ToM was influenced by the language-based measure’s (i.e., ACT Reading, ACT English). This would indicate that the source of the association between the two constructs is largely due to language ability in this sample.

We hypothesized that the path from the math measure would be negligible and that the relationship between ToM and *g* would decrease in the formative*-g* SEMs if the model was indeed language-dependent, suggesting that the relationship between ToM and *g* in this model is influenced by language ability. Specifically, we predicted that even though *g* possibly influences ToM, this does not necessarily mean that ToM is solely driven by general ability.

### 2.1. Method

#### 2.1.1. Design and Subjects

The subjects were 551 students from two large state universities that participated in an experiment on intelligence and cooperation. The data were made available by Freeman et al. (2019). A subset of the data was used by Coyle et al. (2018). The dataset was part of another study on intelligence and cooperation, therefore there were a number of tasks, including ACT tests and ToM tasks, among other measures. Only the measures of interest, ACT and ToM tasks, were included in this study. Only students with data for all three ACT tests and both ToM tasks were included in the analysis. The total number of subjects after excluding missing data was 278. The mean age of the sample was *M* = 19.48 (*SD* = 2.01); 181 (65%) were female.

#### 2.1.2. Procedure and Materials

Three ACT subtests (Math, English, Reading) were used to obtain a *g* factor, and two ToM tasks (RMET and SSQ) were used for the ToM factor. Participants completed both ToM tasks while their ACT scores were obtained from their academic records.

##### ACT

ACT scores ranged from 0–36 and were divided into three subtests: Math, English, and Reading. The ACT is a standardized test used for college admissions in the US and includes two verbal tests (ACT English and ACT Reading) and a Math test (ACT Math). All subtests were included in the dataset (Freeman et al. 2019) and ACT scores were obtained from students’ academic records. See Table 1 for descriptive statistics.

##### Reading the Mind in the Eyes Test (RMET)

RMET scores ranged from 0 to 36 in a discrete fashion. The RMET (Baron-Cohen et al. 2001) presents pictures of people’s eyes (shown one at a time). Each pair of eyes shows a different emotion. Four possible verbal descriptions of emotions are shown next to each pair of eyes (e.g., sad, contemplative, scared, depressed). Participants must respond by selecting the most appropriate emotion that the eyes convey by choosing one of the four responses. Each question answered correctly is a point and the total sum was calculated. See Table 1 for descriptive statistics.

##### Short Stories Questionnaire (SSQ)

SSQ scores ranged from 0 to 27 in a discrete fashion. In the original task by Dodell-Feder et al. (2013) participants must read several short stories about different characters. In each story, a socially inappropriate incident might happen (i.e., incorrectly assuming someone’s age). Then, participants must infer the mental states of the characters (i.e., how they felt, what they thought). Participants are also presented control questions that involve responding to general story comprehension questions. Correct responses were awarded a point and the total sum was calculated. See Table 1 for descriptive statistics.

### 2.2. Results

All analyses were conducted on the data from subjects that completed all three subtests of the ACT, the RMET and the SSQ. The full script is available at: https://osf.io/ke7fc/. From the original dataset, 284 subjects had data for ACT, but only 278 had data for all ACT and ToM tasks. Descriptive statistics for each variable can be found in Table 1 and correlations among variables are presented in Table 2. In this study, ACT scores were conceptualized as a concept largely representative of *g.* While *g* and ACT are strongly correlated (Coyle and Pillow 2008), we consider that they are likely two separate but related constructs. However, for the purpose of replicating and further exploring the results reported by Coyle et al. (2018), in the study we conceptualize ACT as *g.*

Normality tests were conducted to assess whether the data was univariate and multivariate normal. Although for univariate normality skewness and kurtosis values were all within an acceptable range (<±1.00; see Table 1), the assumption for multivariate normality was not met (*HZ* = 1.22, *p* < 0.001). A Bartlett’s test for sphericity produced a significant result, indicating that the variances of the variables were not homogeneous. In addition, we conducted a Kaiser-Meyer-Olkin test to assess sampling adequacy. The result showed that the variance among and within variables was adequate to perform factor analysis (all factors were >0.60). Despite the lack of multivariate normality, we conducted the reflective *g* model analyses using a maximum likelihood (ML) estimator to replicate the original model by Coyle et al. (2018). However, the novel formative *g* models were all conducted using a more appropriate extraction method for non-normal data (i.e., robust maximum likelihood; RML).

#### 2.2.1. Reflective-g Analyses

To address the adequacy of the fit for all of the models conducted, we followed Kline’s model fit indices recommendations. Specifically, Kline indicates that adequate models should have a chi-square to degrees of freedom ratio lower than 2, a Comparative Fit Index (CFI) greater or equal to 0.90, a Standardized Root Mean Square Residual (SRMR) lower or equal to 0.08, and a Root Mean Square Error of Approximation (RMSEA) between 0.05 and 0.10 (Kline 2015).

A confirmatory factor analysis (CFA) with reflective *g* was conducted first to assess the measurement model reported by Coyle et al. (2018). Because the reflective-*g* CFA and SEM were equivalent, only the SEM is reported here. The results of the CFA showed that the model presented an adequate representation of the structure of the data (In the CFA, a model where the *g* construct was correlated with the ToM construct was specified and manifest variables within each construct were correlated. Maximum likelihood was chosen as an estimator to replicate the analyses of the original measurement model by Coyle et al. (2018). Fit indices showed that the model had a good fit (x^2^= 1.72, df = 4, CF(TLI) = 1.00(1.01), AIC = 7571.20, BIC = 7611.10, SRMR = 0.014), with adequate correlation between *g* and ToM (0.57). To further examine the model presented by Coyle et al. (2018), we conducted an SEM model next.

**Figure 1:**
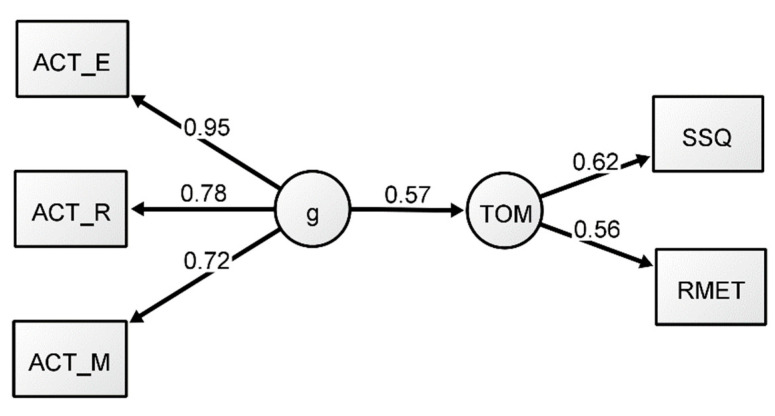
Reflective-*g* SEM Model 1. All coefficients presented are standardized.

A structural equation model (SEM) with reflective *g* was conducted to assess the hypothesis that ToM can be largely explained by *g* (Model 1). Maximum likelihood was again chosen as an estimator to replicate the original analysis from Coyle et al. (2018). The underlying measurement model was specified using the same structure as the previous CFA except a predictive path was added from *g* to the ToM factor instead of a correlational path (see Figure 1). Fit indices for Model 1 (Table 3) demonstrated a good fit to the data. All indices were within the recommended range. The predictive path from *g* to ToM was adequate (0.57), but slightly lower than the results of Coyle et al. (2018). Consistent with the CFA, all factor loadings were satisfactory (see Figure 1). Overall, the model replicated the results reported by Coyle et al. (2018). However, to further examine the relationship between ToM and intelligence, we next conducted a series of analyses from the perspective of formative *g*.

#### 2.2.2. Formative-*g* SEM Analyses

To explore whether a formative *g* approach would provide a better representation of the data, an SEM was conducted with an identical structure to Model 1, except the *g* factor was formative rather than reflective. This theoretical change assumes that *g* is not the underlying cause of the variance common to the manifest variables, but rather it emerges from the manifest variables’ shared variance. It should be noted that CFAs are not conducted on formative models, because measurement models must be reflective in nature. Thus, an SEM was conducted (Model 2) with a predictive path added from *g* to the ToM factor. Unlike the reflective *g* models, the formative *g* models were conducted using an RML extraction method, which is an appropriate technique for data that is not multivariate normal (Finney and DiStefano 2013).

**Figure 2:**
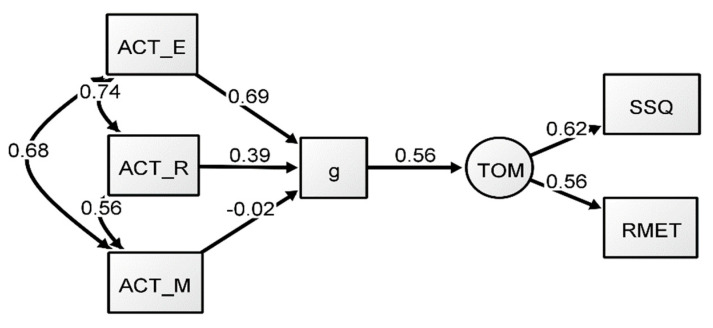
Formative-*g* SEM model (Model 2). All coefficients presented are standardized.

The results of Model 2 can be found in Table 3. Generally, the formative *g* models provided a good fit, similar to the previous reflective *g* models. For Model 2, the predictive path from *g* to ToM showed a coefficient of 0.56, largely identical to that of the reflective-*g* model (Model 1). However, in terms of factor loadings, there were differences compared to those reported in Model 1. The language manifest variables had adequate estimates, but the loading path of the ACT Math test was negligible, indicating that this task seemed to be contributing little to no variance to the emergent *g* factor (see Figure 2), whereas ACT English seemed to provide the largest contribution. The results of the formative-*g* model suggest that the inclusion of the Math variable had little effect on the predictive path between ToM and *g* in this model (in addition, see Appendix A). This indicates that math did not contribute significantly to the variance in the model, suggesting that the language variables seemed to be an important predictor of ToM in this model. These results point to the conclusion that the predictive path from *g* to ToM is influenced by the verbal tasks in this model, especially by ACT English.

#### 2.2.3. Additional Formative-*g* SEM Analyses

**Figure 3:**
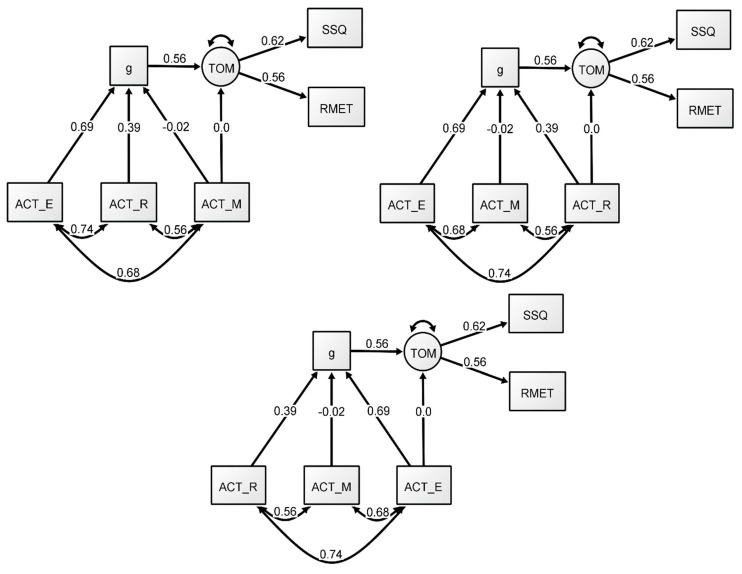
Formative-*g* SEM models with additional paths. All coefficients presented are standardized.

The results of the formative model in the previous analyses showed that ACT English had the highest relative importance in the relationship between formative-*g* and the ToM factor in this model, whereas ACT math provided little to no contribution. However, these models did not directly address the question of whether each independent sub-test (i.e., English, math, and reading) predicts ToM in this model, without the need for a formative-*g*. To assess this question, we conducted three additional SEMs. The models were estimated in an identical way to Model 2, except that a path from each individual ACT measure to the ToM factor was added to each respective model to assess whether the measures were predictive of ToM independently of *g*. Model fit indices can be found in Table 3 (Models 3–5). As expected, we found no significant predictive direct paths from any of the ACT measures to ToM (see Figure 3), suggesting that individual variables were not directly related to ToM. Overall, just like in the previous formative model (Model 2), the English (Model 3) and Reading (Model 4) measures showed strong loadings through formative-*g*, while the Math measure (Model 5) showed a nonsignificant association. These findings support the model presented above that showed that there is a relationship between ToM and *g,* but that, at least in this model, it is strongly influenced by the verbal ACT tasks. In other words, the *g* extracted in our models is, almost entirely a verbal *g*, therefore any associations among the variables should be explained by the path between *g* and ToM.

#### 2.2.4. Redundancy Analysis

An alternative way to examine the relationship between *g* and ToM using a formative approach that does not require the specification of an overarching construct is redundancy analysis (RDA). RDA is a method that allows the extraction and summary of the variance in a set of variables that can be explained by predictor variables (ter Braak 1994; Legendre and Legendre 1998; Zuur et al. 2007). Specifically, it is a way to summarize variance between outcome variables that are explained by (i.e., are redundant) explanatory variable(s). In other words, the results of this analysis can explain how much variance in the outcome variables is redundant with variance in the predictor variables. Thus, RDA was used to estimate the total variance in ToM that was explained by each of the ACT measures and to examine the influence of each ACT measure on ToM. The RDA model was constructed by running the ToM measures as the response variable and the ACT measures as the explanatory (i.e., predictor) variables. The results showed that only about 11% of the variance in the ToM data was explained by considering all of the ACT variables at once, *F*(3, 274) = 11.14, *p* = 0.001, *R^2^* = 0.10. The results show that Math provided the smallest contribution in terms of variance explained in ToM (VIF = 1.89; Regression Coefficient Axis 1 = −0.001, Regression Coefficient Axis 2 = −0.019), whereas English provided the largest (VIF = 2.87, Regression Coefficient Axis 1 = 0.008, Regression Coefficient Axis 2 = 0.009) and Reading provided a moderate contribution (VIF = 2.24, Regression Coefficient Axis 1 = 0.004, Regression Coefficient Axis 2 = 0.002). The results of the RDA provide further support for the idea that the relationship between ToM and *g* in this model is influenced by the language-based nature of the tasks.

### 2.3. Discussion

Overall, the results provide a different approach to examining the *g* construct, by considering it an emergent property resulting from the common variance among tests, and not the cause of those tests. Generally, the results suggested that the overall model fit of the reflective CFA and SEMs to the data was adequate. However, the formative models suggested that only the language measures contributed to the formative *g*. Additional analyses (see Appendix A) supported this finding: (1) the correlation between *g* and ToM decreased significantly in Model 6 and non-significantly in Model 7 compared to Model 2; (2) the factor loading for ACT Reading increased in Model 6 (0.82) compared to Model 2 (0.39) when ACT English was removed; (3) the factor loading for ACT Math remained low-to-negligible in all models, indicating that it contributed little to no variance to the predictive relationship between *g* and ToM; (4) the removal of the Math variable in Model 8 did not have any effect on the predictive relationship between *g* and ToM or in the model fit; (5) finally, constraining ACT English significantly reduced the fit of the model (Model 6). These findings were in line with the results from the RDA analysis. Specifically, the marginal effects showed that math provided the smallest contribution to explaining the variance in ToM measures.

Although the formative *g* models displayed comparable fit to the data than the reflective *g* models, merely comparing the fit of the models is not adequate in this case, as these models represent distinct philosophical perspectives. Traditional reflective models posit that there is an underlying ability (*g* or general intelligence) that directly causes performance on cognitive tests. When taking on this perspective, anytime there is some amount of shared variance among tasks, *g* can be extracted. In other words, if you go searching for *g*, you will find *g*. Alternatively, the formative perspective suggests that domain general and domain-specific processes overlap such that various cognitive abilities may share common processes, as well as having distinct processes from one another. The shared processes allow for common variance and, therefore, a common factor can be extracted, resulting in a formative *g*. As such, in addition to latent variable modeling, alternative means of modeling should be used to examine these potential shared association amongst all manifest variables that are predicted by the formative approach.

## 3. General Discussion

The main goal of this study was to offer an alternative perspective to reflective theories of general intelligence (*g*) to examine whether formative models can provide a more adequate view of the relationship between ToM and *g*. A second goal of this study was to advocate for theories that propose that intelligence is the result of overlapping general and domain-specific processes whose contribution to a given task can vary, instead of an ability caused by an underlying psychological attribute.

The findings from this study showed that although reflective-*g* SEM models presented an adequate fit, the factor loadings differed from those found in the formative-*g* models. Specifically, we found that the contribution of ACT Math to the relationship between *g* and ToM was negligible, suggesting that Reading and English are important predictors of this *g* model. This was further supported in additional analyses (see Appendix A). Specifically, after removing ACT English, the relationship between ToM and *g* decreased, whereas after removing ACT Math, the relationship between ToM and *g* stayed intact. In addition, the Math ACT test explained little to no variance in any of the formative models. This was specifically noticeable when the predictive path from *g* to ToM did not change when Math was removed from the model. That is, while the full model provided the best fit and explained the most variance, the model with math constrained to zero provided identical fit. This indicates that having only English and Reading as predictors or English, Reading, and Math as predictors did not make a difference in the relationship between ToM and *g.* This reveals that the *g* factor formed from this data is influenced by the language-based tasks and that associations with ToM are likely driven by shared language processes. In addition, the results of the redundancy analysis provided further evidence of the relationship between the ACT measures and the ToM tasks from a formative perspective. After examining the contribution of each independent ACT variable to the variance of ToM, we found that ACT math explained the smallest amount of variance. Overall, the findings suggest that it is likely that ToM and intelligence share certain processes, as shown by the strongest fit of the full formative-*g* model and the significant albeit small contribution of the Math measure in the RDA. However, ToM is also influenced by language ability. Importantly, at least in this model, ToM does not seem to depend on solely *g,* whether reflective or formative.

These findings have important theoretical and statistical implications. Specifically, these results indicate that viewing *g* as a formative construct provides a viable framework to better understanding relationships among cognitive abilities. *g* is not an overarching psychological construct but rather an artifact of an interconnected overlapping network of general and domain specific cognitive processes. This is in line with Process Overlap Theory (POT; Kovacs and Conway 2016) that proposes that both general and specific processes are necessary to respond to individual tasks. However, because general processes are thought to overlap and are necessary to respond to a variety of cognitively demanding tasks, general processes are engaged more often than domain-specific processes. At the same time, domain-specific processes are necessary to respond to a given task. Importantly, this theory assumes that general and specific processes are interactive and always engaged in combination and therefore cannot be partitioned for individual assessment.

In addition, the findings from the formative-*g* models suggest that, at least in this model, the relationship between ToM and *g* is influenced by language-related abilities, therefore examining the relationships among different tasks can provide further insight into the general and specific processes that might be necessary for ToM. For example, while ToM and language-specific *g* tasks were more related in this study (i.e., shared more domain-specific processes), other tasks of general ability (e.g., inhibition) might also be related to ToM. In fact, a large number of abilities seem to contribute to ToM (Schaafsma et al. 2015). Both behavioral and neuroimaging data have shown that there are numerous cognitive and non-cognitive processes that influence ToM, such as inhibiting one’s perspective, creating models of alternative emotional responses, and updating one’s own knowledge, but also perceptual and social processes, such as eye gaze movement, emotion recognition, context, and social attention. Future research should focus on understanding the extent to which the overlap among these and other processes affects ToM performance, rather than focusing on whether one single ability might cause ToM.

The findings in this study are also important for research that focuses on examining the residual variance of intelligence tasks as a measure of domain-specific processes. While some research has found correlations between the residual variance of standardized testing and ability-specific tests (Coyle and Pillow 2008; Coyle et al. 2013), other research has found that residuals have no predictive validity (Ree and Earles 1996). More importantly, it is unclear how this practice fits within formative intelligence theories that propose that processes are interconnected and therefore should not be separated. Specifically, if one is to assume that the positive manifold is the consequence of multiple overlapping processes rather than a single general factor, attempting to extract domain-general variance from these variables would result in data that no longer truly captures a cognitive ability at all.

Further, another implication of the current study concerns ToM theoretical accounts. Specifically, numerous theories have attempted to explain the processes underlying ToM in the last decades (see Schaafsma et al. 2015). Based on behavioral and neuroimaging data, most of these accounts suggest that ToM is likely the result of a number of interdependent subprocesses that rely on both domain-general and domain-specific processes. The results of this study indicate that the ToM tasks employed seem to be poorly correlated and are possibly tapping into different aspects of the same construct, replicating recent findings (Warnell and Redcay 2019). However, the SEM showed a relationship between the *g* reflective composite and ToM, indicating that ToM is likely an independent construct. These findings suggest that future research on ToM should focus on understanding the interrelationships among ToM tasks, improving ToM measurement, and adopting a formative approach that could help better illuminate ToM mechanisms.

More generally, this project proposes alternative approaches to conceptualizing and modeling intelligence without characterizing general ability as the psychological attribute that causes all other abilities. Instead, these results indicate that future research should consider a different perspective: that intelligence consists of a network of different processes that overlap and are engaged differently based on task demands. Similarly, the current results suggest that ToM should not be viewed as an all-or-none ability controlled by an overarching psychological construct. Instead, research should move towards studying and describing the interconnected processes and sub-processes that are engaged and interact when using ToM.

Finally, this study also contributes to the increasing amount of research that focuses on using different approaches to examine the relationships among psychometric, cognitive, and developmental models of cognition. Much early research explored psychometric and cognitive models separately (e.g., see Fried 2020 for further discussion of this issue), leading to a poorer understanding of how theory and measurement mesh while also serving different functions. The study conducted in this paper attempts to encourage research that combines both psychometric and psychological approaches to understand cognition, but with the emphasis that the psychometric models must truly characterize the psychological perspective in order to accurately assess the theory. This approach allows for a better understanding of the extent to which a psychometric model of intelligence truly supports a specific theoretical account. In fact, we propose that it is necessary to examine evidence from both psychometric and psychological accounts for consistency to be able to marry different existing models and theories. We expect that the findings reported in this paper incite more research that demonstrates an understanding that psychological and psychometric models are not equivalent while using this combined approach to make better inferences. Specifically, research in the field must ensure the statistical/psychometric models that are used to examine a theoretical perspective (and the associated psychological model) must match the specific theory they are trying to test (Fried 2020). Future research should consider the philosophical perspectives underlying theoretical accounts and corresponding psychometric models to ensure that their statistical and psychometric techniques are consistent with, but distinguished from, the cognitive, developmental, or other models they propose and test.

## Figures and Tables

**Table 1:** Descriptive Statistics.

	*N*	*M* (*SD*)	Skewness	Kurtosis	Min	Max
ACT Math	278	22.56 (4.39)	0.17	−0.56	13	35
ACT English	278	23.96 (5.31)	5.31	−0.26	10	36
ACT Reading	278	24.88 (5.54)	0.01	−0.75	12	36
SSQ	278	18.41 (3.42)	−0.63	0.58	5	27
RMET	278	27.50 (3.54)	−0.79	0.98	11	34

**Table 2:** Correlations with confidence intervals.

Variable	1	2	3	4
ACT Math				
ACT English	0.68 **			
	[0.61, 0.74]			
ACT Reading	0.56 **	0.74 **		
	[0.47, 0.64]	[0.68, 0.79]		
RMET	0.20 **	0.30 **	0.28 **	
	[0.09, 0.31]	[0.19, 0.41]	[0.17, 0.38]	
SSQ	0.24 **	0.33 **	0.31 **	0.35 **
	[0.12, 0.35]	[0.22, 0.43]	[0.20, 0.41]	[0.24, 0.45]

Values in square brackets indicate the 95% confidence interval for each correlation. ** indicates *p* < 0.01.

**Table 3:** Model Fit Indices for the main models (Model 1 and Model 2) as well as for the additional models. Models 6–8 are described in Appendix A.

Fit Indices	χ^2^	*df*	CFI (TLI)	RMSEA	AIC	BIC	SRMR
				(*p*-Value)			
Model 1: Reflective-*g* SEM	1.72	4	1.00 (1.01)	0.001 (0.93)	7571.20	7611.10	0.014
Model 2: Formative-*g* SEM	0.10	2	1.00 (1.02)	0.001 (0.98)	7573.59	7620.74	0.003
Model 3: Formative-*g* SEM (added English path)	0.096	5	1 (1.024)	0.00 (1.00)	7567.585	7603.862	0.003
Model 4: Formative-*g* SEM (added Reading path)	0.093	5	1 (1.024)	0.00 (1.00)	7567.585	7603.862	0.003
Model 5: Formative-*g* SEM (added Math path)	0.097	5	1 (1.024)	0.00 (1.00)	7567.585	7603.862	0.003
Model 6: Formative-g SEM (English constrained)	8.29	3	0.99 (0.96)	0.08 (0.17)	7580.37	7623.91	0.03
Model 7: Formative-g SEM (Reading constrained)	3.32	3	0.99 (1.00)	0.02 (0.63)	7575.22	7618.75	0.02
Model 8: Formative-g SEM (Math constrained)	0.11	3	1.00 (1.02)	0.00 (0.99)	7571.60	7615.13	0.003

## Data Availability

Data of this study were published by Freeman et al. (2019) and not currently available. R scripts and data matrix can be found at: https://osf.io/ke7fc/.

## Appendix A

In addition to the main analyses, we assessed whether the language-based tests of the ACT were responsible for the majority of the association between ToM and intelligence. For this, we conducted three additional models, in which one of the manifest variables underlying ToM had their factor loading constrained to zero. Model 6 (Reading constrained to 0), Model 7 (English constrained to 0) and Model 8 (Math constrained to 0) were identical to Model 2, except that one of the ACT variables’ factor loading was constrained to zero in the analysis. The results of the additional models are in Table 3. In Model 6, the factor loading from the predictive path between *g* and ToM decreased, from 0.56 (Model 2) to 0.51 (Model 3). Importantly, by removing the ACT English test, the factor loading for ACT Math was still low and almost negligible. In Model 7, the factor loading from the predictive path between *g* and ToM had again a slight decrease, from 0.56 (Model 2) to 0.54 (Model 4). The factor loading for the ACT Math score remained negligible in this model. Finally, in Model 7 the factor loading from the predictive path between *g* and ToM presented the same estimate as in Model 2 (i.e., model with all variables), that is, 0.56, while the other two manifest variables presented adequate factor loading values. Although, we could not formally compare the constrained models because they had equal degrees of freedom, each of the models were compared to the original Model 2. All models had equivalent fit to the full model, except for Model 6 (i.e., model with the factor loading for ACT English constrained to zero), which had significantly worse fit than the full model (Model 2; Δχ^2^(1) = 7.78, *p* = 0.005). This indicates that when English does not contribute to the formative-*g* factor, the model is significantly less accurate at describing the data. However, these results should be interpreted with caution as the limited number of variables used to assess each of these two broad constructs can hinder the interpretation of the models.
